# Factors associated with dislocation after bipolar hemiarthroplasty through an (antero-)lateral approach in elderly patients with a femoral neck fracture: a retrospective cohort study with a nested case–control subanalysis of radiographic parameters

**DOI:** 10.1007/s00068-022-01918-x

**Published:** 2022-03-30

**Authors:** Johannes Karl Maria Fakler, Markus Rositzka, Nicolas Schopow, Andreas Roth, Dirk Zajonz, Mohamed Ghanem, Christian Kleber, Georg Osterhoff

**Affiliations:** 1 Department of Orthopedic, Trauma and Plastic Surgery, University Hospital of Leipzig, Liebigstr. 20, 04103 Leipzig, Germany; 2 Department of Diagnostic and Interventional Radiology, University Hospital of Leipzig, Liebigstr. 20, 04103 Leipzig, Germany; 3 Department of Trauma, Hand, Reconstructive and Spine Surgery, Klinikum Passau, Innstr. 76, 94032 Passau, Germany; 4 Department of Orthopedic Trauma and Reconstructive Surgery, Zeisigwaldkliniken Bethanien Chemnitz, Chemnitz, Germany

**Keywords:** Hip fracture, Hemiarthroplasty, Dislocation, Radiographic analysis, Comorbidity, Complications

## Abstract

**Introduction:**

Dislocations of hip hemiarthroplasty (HHA) are serious complications. The aim of the study was to identify clinical aspects and radiographic parameters of the hip that put patients at risk for dislocation after HHA for femoral neck fractures.

**Methods:**

This retrospective analysis included elderly patients with a femoral neck fracture treated with a HHA. A lateral (90.7%) and an anterolateral (9.3%) approach was applied. On pelvic radiographs, a nested-controlled analysis was performed. Two control patients were matched to one patient suffering a dislocation with respect to age, sex, and body-mass-index (BMI).

**Results:**

In 527 HHA, 10 dislocations (1.9%) were identified. In the dislocation group (DG), all patients were female (100% vs. 73.5%, *p* = 0.071). No significant differences between the DG and the control group (CG) were found with respect to age, body-mass-index (BMI), ASA Score, routine laboratory parameters, and comorbidity. Radiographic analysis revealed a smaller center edge angle (CEA, 39.0, IQR 33.0–42.5 vs. 43.0, IQR 41.0–46.0, *p* = 0.013), a more varus neck-shaft angle (NSA, 130.0, IQR 125.8–133.5 vs. 135.0, IQR 134.0–137.0, *p* = 0.011) of the contralateral side and a higher femoral head extrusion index (FHEI) in the DG (FHEI, 11.5, IQR 9.8–16.3 vs. 2.0 IQR 0.0–9.0, *p* = 0.003). In addition, a greater trochanteric fracture was associated with an increased likelihood for HHA dislocations (30.0% vs 6.0%, *p* = 0.022).

**Conclusion:**

A smaller radiographic center edge angle, a more varus neck-shaft angle of the contralateral side, a higher femoral head extrusion index and intraoperative fractures of the greater trochanter are associated with an increased risk of HHA dislocation.

## Introduction

Hip fractures are common injuries in elderly patients implicating a major burden to healthcare systems worldwide. It is estimated that the incidence of hip fracture will rise from 1.66 million in 1990 to 6.26 million by 2050 [1]. For displaced femoral neck fractures in elderly patients, that are most commonly affected, arthroplasty is the treatment of choice [2]. However, there is still an ongoing debate whether the optimal treatment in these cases is hip total versus hip hemiarthroplasty. Functional results are reported to be in favor of total hip arthroplasty (THA), but the advantage seems to be below [3] or at [4] the threshold for a minimal clinically relevant difference. On the other hand, hip hemiarthroplasty (HHA) is associated with a lower blood loss [3–5] and a shorter duration of the surgical procedure [3, 5, 6]. Regarding surgical complications and the risk of secondary procedures THA and HHA show similar results within the first 2 years after index operation [3, 5–7]. However, the risk of hip dislocation is 2- to 3-times higher after THA compared to HA in these patients [3, 4, 8]. Biomechanically, higher hip stability after HHA is explained by its large head to neck ratio. Dislocation occurs beyond the jump distance which is a femoral head displacement out of the acetabulum greater than half of its diameter [2]. Hence, dislocation rates after HHA are reported to be low ranging from 0 to 2.4% by most studies [3, 5, 8–10] while some report moderate to high rates of 6–11% [11–13]. Irrespective of the incidence, hip dislocation represents a potentially devastating complication with a reduced quality of life [14], a 2.5-fold increased mortality at 3 months [15] and a total mortality of up to 65% after 6 months [9].

Multiple patient- and surgery-related risk factors are discussed for hip dislocation after HHA. A posterior surgical approach [16, 17] and a reduced postoperative femoral offset [12, 18] seem to be associated with higher dislocation rates. On the patients’ side, mental impairment [12, 19] and anatomical aspects, as a reduced center edge angel (CEA) of the acetabulum [12, 18], are reported to promote dislocation. With regard to hip anatomy measured by radiologic parameters on plain pelvic radiographs, as coverage of the femoral head or reconstruction of lateral femoral offset, only limited information is available. Therefore, the primary aim of this study was to analyze radiologic parameters that potentially identify patients at risk for dislocation after HHA. In addition, further clinical risk factors related to patients and surgery were analyzed.

## Methods

### Ethics

The study was approved by the local Ethics Committee at the University of Leipzig 2021-04-13 (ref. 144/21-ek). The study was registered retrospectively with the ResearchRegistry (UIN: researchregistry6812). The procedures used in this study adhere to the tenets of the Declaration of Helsinki.

### Patient selection

For this retrospective cohort study, all consecutive patients primarily treated with bipolar HHA for a femoral neck fracture between July 2010 and May 2020 were included. Patients presenting with a pathologic fracture, prior internal fixation or a neglected fracture older than 4 weeks were excluded. Inclusion criteria were met in 512 patients with 527 HHA.

Follow-up was performed at our out-patient clinic or after re-admission due to surgery- and non-surgery-associated reasons. Patients who did not present in the out-patient clinic or in the hospital were sent a questionnaire 3 months after surgery in which complications were asked for. Median follow-up was 12 (1–24) months and a minimum follow-up of 3 months or until death within 3 months was available in 80.3% of all HHA. In patients with a HHA dislocation, the minimum follow-up was 12 months or until death within 12 months.

### Surgical treatment

Surgery was performed in general anesthesia in all patients. Patients were placed in a supine position and a modified lateral Hardinge approach was applied in 90.7% and an anterolateral approach in 9.3%. The different distribution is explained by a more recent introduction of the anterolateral approach. A bipolar hemiprosthesis (DePuy Synthes, USA) was the standard implant at our institution.

All patients were allowed to fully weight bear and were instructed to use a walker or two crutches, if possible. They were advised to limit hip flexion to 90° and to avoid adduction within the 6 first postoperative weeks. No additional hip precautions as for example hip adduction pillows were applied.

### Radiographic evaluation

For radiographic analysis a nested-controlled analysis was performed. Two control patients without HHA dislocation were matched to one patient suffering a dislocation with respect to age, sex, and body-mass-index (BMI). In both the groups, the same standard stem with a 135° neck-shaft angle of the prosthesis (NSAP) was used in all patients. In one patient with a dislocation, an anterolateral approach was used. For this patient, one control patient with an anterolateral and a second control patient with a lateral approach was matched. All other patients had a lateral approach and were matched with two control patients who also received a lateral approach.

Postoperative standard anteroposterior pelvis with the X-ray beam centered to the pubic symphysis and lateral hip radiographs were digitally acquired using the Picture Archiving and Communication System (Syngo Plaza, Siemens healthineers, Germany). All radiographic measurements were performed by an experienced radiologist using a PACS software (Magic Web VA60C, Visage Imaging, Germany) on a diagnostic color monitor authorized for use in radiology (Coronis Fusion 6MP (MDCC-6530), Barco, Belgium).

**Fig. 1:**
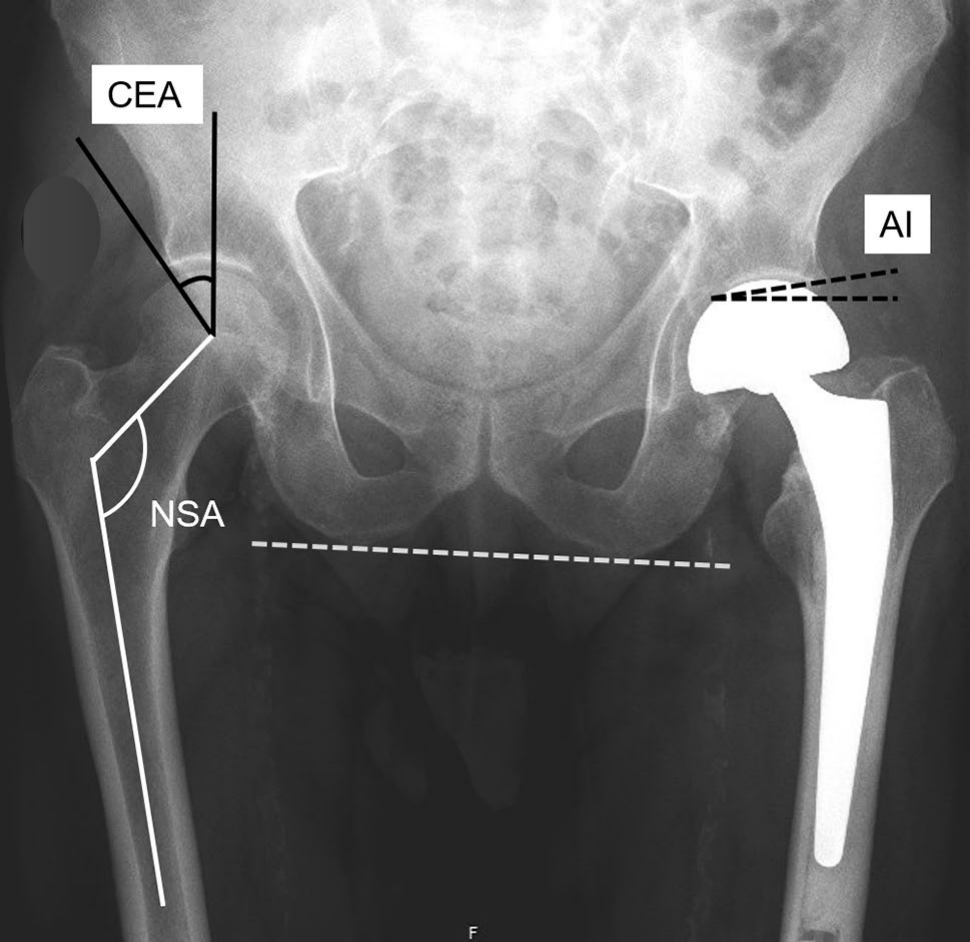
Standard pelvic radiograph with delineated acetabular center edge angle (CEA), neck-shaft angle (NSA) and acetabular index (AI)

**Fig. 2:**
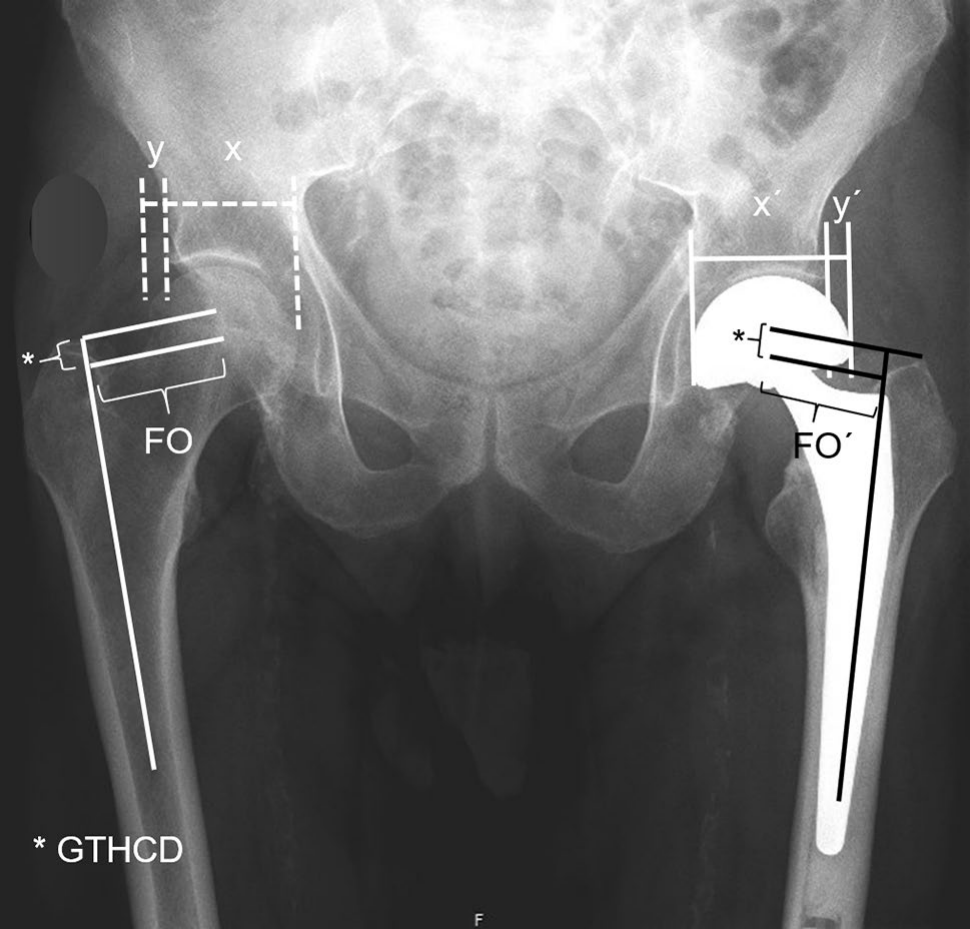
Standard pelvic radiograph with delineated lateral femoral offset of the unaffected side (FO) and after hemiarthroplasty (FO´), femoral head extrusion index [FHEI = *x*/(*x* + *y*)], bipolar head extrusion index [BHEI = *x*´/(*x*´ + *y*´)], greater trochanteric tip hip center distance (GTHCD)

Radiographic parameters analyzed were: center edge angle (CEA) of Wiberg, acetabular index (AI), femoral head diameter, depth-to-width index (DWI), femoral head extrusion index (FHEI), bipolar head extrusion index (BHEI), neck-shaft angle (NSA), lateral femoral offset (FO), and tip of greater trochanter to hip center distance (GTHCD). Except for FHEI and BHEI, all measurements were performed bilaterally (Figs. 1 and 2).

The CEA was measured as the angle between a perpendicular through the hip center and the lateral edge of the acetabulum. The TF/AI was measured as the angle between a horizontal line and a parallel line to the weight-bearing part of the acetabulum. The DWI was calculated by the acetabular depth in relation the acetabular diameter multiplicated by 100. The FHEI (BHEI) describes that part of the femoral (bipolar) head that is not covered by the acetabulum which is the distance from the acetabular edge to the lateral border of the femoral (bipolar) head in relation to the distance between the medial and lateral border of the femoral (bipolar) head multiplicated by 100 [20]. The NSA was measured as the angle between the femoral shaft axis and the longitudinal axis of the femoral feck. Lateral FO was defined as the distance between the femoral shaft axis and the center of the femoral or bipolar head. The GTHCD was measured as the distance between a line at the tip of the greater trochanter perpendicular to the femoral shaft axis and the femoral or bipolar head center.

### Statistical analysis

Continuous variables were analyzed for normal distribution applying the Kolmogorov–Smirnov test in addition to *q*–*q* diagrams. For non-normally distributed parameters, median and interquartile range [25–75th percentile] were used. The Mann–Whitney *U* test was applied for non-normally distributed variables. For categorical comparisons, according to group size, Chi-squared or Fisher’s test were performed. All statistical computations were performed using SPSS version 24.0 (Chicago, IL, USA). *p* values less than 5% were considered as significant.

## Results

In 527 performed bipolar HHA, 10 dislocations were identified (1.9%). Baseline characteristics (Table 1) displayed no significant differences between dislocation group (DG) and control group (CG). Apart from greater trochanteric fractures (GTF), perioperative and surgical parameters did not differ significantly between the groups (Table 2).

**Table 1:** Baseline patient characteristics

	No dislocation (*n* = 517)	Dislocation (*n* = 10)	*p*
Age	83.0 (77.0–89.0)	86.0 (80.8–88.0)	0.502
Female sex (%)	380 (73.5)	10 (100)	0.071
BMI	24.2 (22.0–26.9)	23.4 (20.8–26.9)	0.494
ASA Score			0.439
I	6 (1.2)	0 (0.0)	
II	129 (25.0)	1 (10.0)	
III	370 (71.6)	9 (90.0)	
IV	12 (2.3)	0 (0.0)	
CRP (mg/l)	6.1 (1.7–26.1)	2.7 (1.2–8.6)	0.155
Creatinine (mmol/l)	79.0 (64.0–104.0)	77.0 (67.5–88.0)	0.783
Hemoglobin (mmol/l)	7.7 (6.9–8.4)	7.4 (6.3–7.9)	0.154
Prothrombin time (%)	96.0 (82.0–106.0)	102.0 (73.0–114.5)	0.422
Platelet inhibitors	168 (32.5)	2 (20.0)	0.512
Warfarin	89 (17.2)	3 (30.0)	0.390
NOAC	41 (7.9)	1 (10.0)	1.000
Diabetes	142 (27.5)	2 (20.0)	0.740
Cognitive impairment	139 (26.9%)	3 (30.0%)	1.000
Neuromuscular disease	71 (13.7%)	3 (30.0%)	0.154

Values are given as median with interquartile range (IQR) or as absolute numbers with percentage in parenthesis

*BMI* body-mass-index, *NOAC* novel oral anticoagulants

**Table 2:** Perioperative parameters

	No dislocation (*n* = 517)	Dislocation (*n* = 10)	*p*
Time to surgery (h)	25.0 (16.0–41.0)	16.5 (8.5–33.0)	0.142
Surgery < 24 h (%)	248 (48)	7 (70)	0.209
Duration surgery (min)	72.0 (57.5–85.0)	74.0 (65.8–96.3)	0.439
Day-time surgery (%)	417 (80.7)	7 (70.0)	0.419
Surgery by resident	132 (25.5)	1 (10.0)	0.315
Lateral approach (Hardinge)	469 (90.7%)	9 (90.0%)	1.000
Cemented stem	466 (90.1)	10 (100)	0.684
Greater trochanteric fracture (GTF)	31 (6.0)	3 (30.0)	0.022

Values are given as median with interquartile range (IQR) or as absolute numbers with percentage in parenthesis

In both the groups, no significant differences in terms of radiographical acetabular parameters between fractured and unaffected side could be found. Although no dysplastic hips were identified, the acetabular CEA was significantly smaller in the dislocation group compared to the control group (*p* = 0.013). In accordance, the FHEI was substantially higher in the DG (*p* = 0.003). Correspondingly, the BHEI was also significantly higher in the DG (*p* = 0.006). On the femoral side, a decreased or more varus NSA of the non-operated side was found to be associated with an increased risk of dislocation (*p* = 0.011). The postoperative FO did not differ between the groups (*p* = 0.534) (Table 3). Comparing pre- and postoperative FO demonstrated a minimal reduction by approximately 2 mm in the DG (*p* = 0.246) as well as in the CG (*p* = 0.566). A standard stem with a 135° NSAP was used in all cases. Comparing the preoperative NSA with the NSAP, a significant increase was exhibited only in the DG (*p* = 0.003), but not in the CG (*p* = 0.741).

**Table 3:** Radiographic measurements on standardized pelvic radiographs (matched-pair analysis)

	No dislocation (*n* = 20)	Dislocation (*n* = 10)	*p*
Center edge angle (CEA)	43.0 (41.0–46.0)	39.0 (33.0–42.5)	0.013
Acetabular index (AI)	5.0 (2.0–7.8)	4.5 (2.0–10.0)	0.735
Femoral head diameter (mm)	46.0 (45.0–46.0)	44.0 (42.0–46.5)	0.168
Depth-to-width index (DWI)	51.0 (47.0–57.0)	48.0 (44.0–51.5)	0.223
Femoral head extrusion index (FHEI) unaffected side (mm)	2.0 (0.0–9.0)	11.5 (9.8–16.3)	0.003
Neck shaft angle (NSA) unaffected side	135.0 (134.0–137.0)	130.0 (125.8–133.5)	0.011
Lateral femoral offset (FO) unaffected side (mm)	37.0 (33.0–39.0)	35.5 (33.8–37.8)	0.625
Greater trochanter hip center distance (GTHCD) unaffected side (mm)	− 2.0 (− 5.0 to − 1.0)	− 4.5 (− 7.3 to − 1.5)	0.388
Bipolar head diameter (mm)	47.0 (46.0–48.0)	46.0 (44.0–48.5)	0.802
Bipolar head extrusion index (BHEI)	8.0 (4.0–17.0)	25.0 (13.0–32.5)	0.006
Postoperative lateral femoral offset (FO) (mm)	35.0 (33.0–39.0)	34.0 (33.0–35.5)	0.534
Postoperative greater trochanter hip center distance (GTHCD) (mm)	− 2.0 (− 4.0 to 0.0)	1.0 (− 6.5 to 5.0)	0.380

Values are given as median with interquartile range (IQR) or as absolute numbers with percentage in parenthesis

Early dislocation within 6 weeks occurred in 8/10 of patients with a dislocation without a prior fall. In two patients, a dislocation was asserted after a fall 7 and 10 weeks after surgery. Recurrent dislocation was present in 4/10 of patients. These patients underwent revision surgery to total hip arthroplasty (THA) with a cemented cup. In 4/10 of patients, closed reduction was not possible. Revision included open reduction with change of the bipolar head in two patients, change of the prosthesis stem due to malrotation in one patient, and revision to THA with a cemented cup in one patient. Two patients were treated with closed reduction with no further dislocations. After revision surgery, three patients developed a periprosthetic infection warranting additional interventions. One patient was managed with exchange of the bipolar head, jet-lavage and antibiotics, another patient with a two-staged revision THA and one patient with removal of the THA and girdlestone arthroplasty. However, 1-year mortality was not increased in patients suffering a dislocation (DG 22.2% vs. CG 20.0%, *p* = 1.000).

## Discussion

The primary aim of the study was to identify clinical aspects and radiographic parameters of the hip that put patients at risk for dislocation after HHA for femoral neck fractures.

In the recent literature, the reported rate of dislocation after HHA in patients treated for femoral neck fractures ranged from 0 to 11% [5, 13, 21]. This is in line with our study that found a rate of 1.9%. Risk factors for dislocation of a HHA can be divided in those related to patient characteristics or those related to surgical aspects. Radiographical parameters on plain pelvic radiographs address both and may help to identify patients who have an individual anatomical risk or support postoperative analysis of potential surgical failure.

In our study, the CEA was significantly smaller in the DG compared to the CG which is in line with other studies [12, 13, 18, 22]. Only one study clearly found no influence of the CEA on dislocation rates. Noteworthy, this study exhibited a considerable change (> 10%) of the postoperative FO in more than 50% of HHA. This relevant FO difference was also identified as a significant risk factor for dislocation in this study, and subsequently, might have masked a potential effect of the CEA [23].

Another radiographical risk factor for dislocation in our study was an increased FHEI as well as the BHEI corresponding with a reduced acetabular coverage. The BHEI has been confirmed as a risk factor for dislocation by Zhang et al. [22]. In opposition, Kizkapan et al. [24] did not identify FHEI or postoperative BHEI as a risk factor for dislocation. THA has the advantage of correcting a shallow acetabulum and adjusting acetabular inclination and version which is not possible with HHA. Thus, identifying morphological acetabular risk factors on preoperative X-rays may aid the surgeon in deciding when to use total over hemiarthroplasty and to reduce dislocation rates.

The NSA on the operated side was comparable between groups which was also described by others [12, 18, 24]. However, the NSA of the unaffected side differed significantly and displayed lower values in the dislocation group. Since a standard 135° stem was used in all HHA with a dislocation, it can be concluded that preoperative NSA was not addressed correctly in our study. Postoperative FO did not influence dislocation rate in our analysis. No difference of postoperative FO in dislocated and not dislocated HHA was also confirmed by Zhang et al. [22], whereas others demonstrated an association between a smaller postoperative FO and dislocation [12, 13, 18, 24, 25]. An explanation for these diverging findings may be that FO of the unaffected side did not differ between groups and postoperative FO was restored quite well with only marginal, not significant differences to the contralateral unaffected side in our study.

Most of the few studies that analyzed radiographical parameters on plain radiographs of the pelvis presented substantial higher dislocation rates ranging from 6 to 11% [12, 13, 18, 24, 25]. One reason might be that the majority of these studies applied a posterolateral surgical approach for HHA [13, 18, 24]. The posterolateral approach tends to higher dislocation rates compared to anterior approaches [15, 16, 23, 26]. One study with a posterolateral approach found a comparatively low dislocation rate of 3% [22]. In contrast to the other studies using a posterolateral approach [13, 18, 24], this study found no difference of postoperative FO between dislocated and not dislocated HHA. Only one study that analyzed radiographic parameters exclusively applied an anterolateral approach [25]. They reported a dislocation rate of 6%, comparable to those with a posterolateral approach [13, 18, 24]. Interestingly, this study demonstrated a significantly higher FO discrepancy [25]. It might be hypothesized that restoration of anatomic FO is more important than the chosen surgical approach with regard to dislocation rate after HHA.

A higher rate of HHA dislocation was reported in patients in which surgery was delayed beyond 24 h [18, 21]. A possible explanation might be that surgery within 24 h might not always allow an optimal arrangement of the surgical team. However, experience of the surgeon did not influence dislocation frequency in our study as in others [21, 23].

Intraoperative GTF in HHA for femoral neck fractures potentially adds to hip abductor weakness and hip instability. Consequently, limping, poor clinical outcome and hip dislocation may be associated with GTF [27, 28]. Following HHA for femoral neck fractures, GTF occurred in up to 6% [29, 30]. Effectively, our study ascertained a significant association between a GTF and dislocation.

Apart from anatomical issues on the patients’ side, cognitive impairment and neurologic disease with peripheral deficiencies may be associated with postoperative HHA dislocations due to incompliance and muscular insufficiency, imbalance, or contracture. In patients with HHA dislocation, the percentage of patients with dementia was 3-times higher compared to patients without a dislocation [12, 25]. Our study revealed a dislocation rate of approximately 2% in both cognitive impaired and healthy patients, which is in line with other study results that did not identify mental dysfunction as a risk factor for dislocation after HHA [18, 21]. We and others [31] also found no influence of neurologic diseases that affect muscular function at the hip as sequelae of stroke or Parkinson’s disease.

Mortality at 1 year might be increased after HHA dislocation due to potentially necessary additional surgery, prolonged immobility and complications associated with revision surgery in this vulnerable geriatric patient cohort. In fact, earlier studies reported a high mortality rate of up to 65% within the first 3–6 months after surgery [9, 15]. In contrary, other studies exhibited only a tendency towards a higher 1-year mortality [12, 13] or could not demonstrate any impact of HHA dislocation on 1-year mortality at all as shown by us and Salem et al. [21].

This study is limited by the retrospective design and a low number of dislocations inherently associated with an (antero-)lateral approach. A nested case–control subanalysis for radiographic parameters was performed which is advantageous over a full cross-sectional cohort design and provides equal accuracy when actual disease or complication prevalence in subjects is low [32]. Due to the low number of complications, subgroups of single and recurrent dislocations were not investigated. Thus, more detailed information about HHA dislocation may have been missed. Second, cognitive deficiencies were assessed by medical records on admission and not quantified using standardized questionnaires. Third, experience of the surgeons was graded by an educational level rather than the annually performed number of hip arthroplasties. Fourth, it is not possible to address version of the acetabulum and torsion of the implant stem accurately on two-dimensional radiographs which are relevant factors in HHA dislocation and might have influenced results. Finally, two different approaches, the lateral and anterolateral approach, were used which may add to some bias.

The results of this study suggest that a smaller CEA, a higher FHEI and a smaller or more varus FNA not addressed by correct choice of an implant stem with a corresponding NSA are radiographic risk factors for dislocation in bipolar HHA through an (antero-)lateral approach. Identifying these morphological parameters on preoperative radiographs which can be addressed by THA and correct choice of implant components may reduce the rate of HHA dislocations. GTF were also associated with an increased risk for HHA dislocation. This again underlines the need for a careful surgical approach to these procedures.
